# Right and Left Coronary and Conus Arteries Originating from Three Separate Ostia in the Right Valsalva Sinus in a Japanese Cadaver: A Case Study with Literature Review

**DOI:** 10.3390/medicina60050730

**Published:** 2024-04-28

**Authors:** Daisuke Kiyoshima, Osamu Tanaka, Hayato Terayama, Ning Qu, Kenta Nagahori, Yoko Ueda, Masahito Yamamoto, Kaori Suyama, Shogo Hayashi, Kou Sakabe

**Affiliations:** 1 Department of Anatomy, Division of Basic Medical Science, Tokai University School of Medicine, 143 Shimokasuya, Isehara-si 259-1193, Kanagawa, Japan; kiyoshima@tokai.ac.jp (D.K.); samasot1@gmail.com (O.T.); quning@tokai.ac.jp (N.Q.); nk761111@tsc.u-tokai.ac.jp (K.N.); ueda4989@gmail.com (Y.U.); yamamotom@tsc.u-tokai.ac.jp (M.Y.); suyama@is.icc.u-tokai.ac.jp (K.S.); sho5-884@umin.ac.jp (S.H.); 2 Department of Anatomy, Tokyo Medical University, 6-1-1 Shinjuku, Shinjuku-ku, Tokyo 160-8402, Japan; 3 Department of Environmental Preventive Medicine, Yamada Bee Company, Inc., Center for Preventive Medical Sciences, Chiba University, 1-33 Yayoicho, Inage-ku, Chiba-si 263-8522, Chiba, Japan

**Keywords:** cadaver, left coronary artery, conus artery, anatomical variants

## Abstract

A rare case of an anomalous location of the orifice of the coronary artery was found in a 99-year-old male cadaver undergoing routine dissection. The presence of the right coronary artery (RCA), left coronary artery (LCA), and conus artery (conus branch) originating from the right Valsalva sinus are the characteristic findings of this case. Then, the LCA passed through the aorta and the pulmonary artery. The LCA and RCA branches were normal. These findings are useful for future surgical procedures, including cardiac catheterization.

## 1. Introduction

The heart is supplied by two coronary arteries (left and right) and their branches; these vessels arise from the right and left sinus of Valsalva as two branches of the ascending aorta. The left coronary artery (LCA) is generally divided into two main branches: the left anterior descending (LAD) and the left circumflex (LCx). The LAD branches into the diagonal branch (DB), whereas the LCx branches into the obtuse marginal branch, posterolateral branch (PL), and posterior descending branch (PD). The right coronary artery (RCA) begins at the right aortic sinus and goes along the coronary sulcus. During its course, it produces the sinus node branch (SN), conus branch (CB), right ventricular branch (RVB), acute marginal branch (AM), atrioventricular node branch (AVM), and posterior descending branch (PD) [1,2]. Coronary artery anomalies are some of the most confusing and neglected topics in cardiology [3].

Congenital malformations accounting for 2.2% of congenital heart malformations are anomalies of coronary artery origins and their associated runs [4,5]. Anomalies of the coronary artery correlate with sudden cardiac death after exercise and angina [4]. Autopsy examinations show that coronary artery abnormalities are the second most common, and hypertrophic cardiomyopathy is the most common cause of sudden cardiac death during strenuous exercise in young athletes (aged <35 years) [6]. Therefore, the morphology of coronary artery anomalies should be examined. However, to the best of our knowledge, several studies were conducted on Westerners, and only a few Japanese reports were conducted. Furthermore, most reports of coronary artery abnormalities are based on coronary angiography, and very few reports focus on the detailed morphology of coronary artery abnormalities. Here, we report a rare case of RCA, LCA, and CB originating from the right Valsalva sinus in a cadaver, along with a literature review.

## 2. Materials and Methods

The cadaver studied was that of a 99-year-old Japanese male (cadaver number: 1879, cause of death: heart failure) who died of cardiac failure. The cadaver was selected from the bodies used for gross anatomy practice at the Tokai University School of Medicine in 2015. This report complies with the research guidelines of the Japanese Association of Anatomists. A cadaver designated (Tokai Daigaku Kentai No Kai) for education or research was used in this study. Informed consent was obtained from the antemortem person by Tokai Daigaku Kentai No Kai. Whether the cause of death was influenced by the heart formation remains unknown. However, no surgical scars were found on the heart. The cadaver was fixed using 10% formaldehyde. Gross dissection was performed using the standard technique. We removed the pericardium, rib, fat, and skin around the chest for observational purposes and carefully examined the structures. The pulmonary artery, pulmonary vein, aorta, inferior vena cava, and superior vena cava were then cut, and the heart was removed. In particular, the coronary arteries were carefully observed.

The coronary artery types in this case were classified according to previous reports [7]. The proximal course of coronary arteries is defined as follows [8]: a course anterior to the pulmonary artery or right ventricular outflow tract is designated as “are pulmonary”, a course posterior to the aorta as “retro aortic”, a course between the great vessels as “interarterial”, and a course through the interventricular septum as “septal”. Another name for this case is the anomalous aortic origin of the left main coronary artery (AAOLCA). The running pattern of the left coronary artery is classified into four types with the same running pattern as the proximal course [9,10]. They are dorsal running of the aorta (retroaortic course: AAOLCA-RA), intervascular running (interarterial course: AAOLCA-IA), ventral running of the right ventricular outflow tract (anterior free wall (prepulmonic) course: AAOLCA-AF), and running within the right ventricular outflow tract within the conal septum (intraseptal (transeptal/intramyocardial/tunneled/intra-conal) course: AAOLCA-IS) [9,10]. The proximal course classification of vessels is associated with heart disease [11,12]. The conus branch was designated “the conus artery (CA)” if it branched from the sinus of Valsalva, and “conus branch (CB)” if it branched from the RCA or LCA. For surgical procedures such as coronary artery grafting and coronary artery bypass grafting, the angles of the ascending aorta and each of the LCA, CB, and RCA were measured [13].

We used the keywords “left AND coronary AND artery AND right AND valsalva AND sinus” for the right sinus of Valsalva with three separate ostia and “left AND coronary AND artery AND right AND valsalva AND sinus” for the conus branch as search terms. Reference searches were conducted using Scopus (https://www.elsevier.com/ja-jp/solutions/scopus accessed on 3 April 2024), and only English literature was collected.

## 3. Results

### 3.1. Case Report

**Figure 1:**
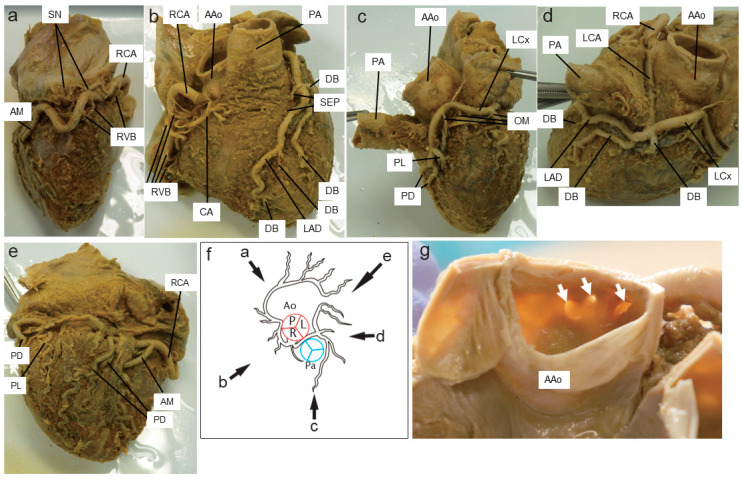
Left and right coronary arteries and branching vessels are observed from various directions: (**a**–**e**) in the figure demonstrate each aspect. Schematic (**f**) shows the location of the coronary artery observed in (a–e). Left and right coronary arteries and branching vessels are shown in the schema (**f**). The origins of the three vessels in the right sinus of Valsalva are indicated by white arrows in (**g**). AAo, ascending aorta; PA, pulmonary artery; RCA, right coronary artery; SN, sinus node branch; RVB, right ventricular branch; AM, acute marginal branch; CA, conus artery; LAD, left anterior descending artery; DB, diagonal branch; SEP, septal perforating branch; LCx, left circumflex artery; OM, obtuse marginal branch; PL, posterolateral branch; PD, posterior descending branch; LCA, left coronary artery.

The RCA entered the coronary sulcus through the anterior right heart ear and branched left one sinuatrial nodal branch (Figure 1a) and five right ventricular branches (Figure 1b). It then turned right and reached the posterior aspect of the heart, branched one acute marginal branch to the right, and descended with two posterior descending branches (Figure 1e). The angle between the RCA and the ascending aorta was 75 degrees.

CA was distributed in the right ventricular outflow tract, and no anastomosis with other arteries could be identified (Figure 1b). The angle between the CA and the ascending aorta was 70 degrees.

The LCA formed an acute angle from the aortic origin, passed through the aorta and the pulmonary artery, and then branched into the anterior interventricular (LAD) and left circumflex (LCx) branches (Figure 1d). The proximal course of the coronary arteries was “interarterial.” The length of the interarterial course of the LCA was 3.67 cm. The LCA type was “AAOLCA-IA”, although the CB originated from the right sinus of Valsalva (“AAOLCA-IA with CA”). The angle between the LCA and the ascending aorta was 50 degrees.

The LAD had two septal perforating branches (SEPs) branching to the right and four more DBs branching to the left while descending the interventricular groove toward the apex (Figure 1b).

LCx curved leftward at the interventricular groove (coronary sulcus) and reached the posterior surface of the heart, whereas three obtuse marginal branches branched off into the left ventricle (Figure 1c). The LCx then traveled through the interventricular groove and branched to posterior descending branches to the left atrium and posterolateral branches to the posterior wall of the left ventricle (Figure 1d).

The RCA, CA, and LCA were open through the right Valsalva sinus (Table 1). The LCA was separated into the anterior interventricular branch and the circumflex branch. In terms of outer diameter, the RCA, CA, and LCA ostia were 4.5 mm, 1.0 mm, and 2.5 mm, respectively (Figure 1g). The distance from the sinotubular junction (STJ) to the origin of the coronary artery was 8.4, 5.1, and 6.8 mm for the RCA, CA, and LCA, respectively. No branches to the coronary artery were observed from the other Valsalva sinuses.

### 3.2. Reference Reports

Among the 1315 references collected, 14 focused on coronary arteries originating from the right sinus of Valsalva with three separate ostia (Table 1). Most reports on the three separate ostia of the right sinus of Valsalva showed patterns of RCA, LAD, and LCx or RCA, LCA, and CA. All reports used angiography.

References about conus arteries originating from the right sinus of Valsalva were collected in 13 of 23 patients (Table 2). CA originating from the right sinus of Valsalva was found to occur with high probability, with an incidence of 13.7–68.0%. The incidence of one CA originating from the right sinus of Valsalva is 12.6–68%. The incidence of two CAs originating from the right sinus of Valsalva is 0.3–1.1%.

Two reports were similar to the present case. However, the method of coronary artery observation was angiography and not a cadaver. Furthermore, the proximal course of the LCA was other than “interarterial.”

## 4. Discussion

In this paper, we report a rare case of RCA, LCA, and CA originating from the right Valsalva sinus. What is new in this case is that we have observed “interarterial” and “AAOLCA-IA with CA” in a cadaver.

Coronary artery anomalies are incidentally detected during angiography or autopsy. The incidence of angiographic series is 0.6–1.3%, with men more frequently affected [25,38,39,40,41]. The incidence of autopsy series is 0.3% [3,42]. Furthermore, the incidence of strictly coronary anomalies is reported to be 5.6% [25,39]. In any event, coronary artery anomalies are generally rare. The anomalous origin of the left main coronary artery from the right sinus of Valsalva represents a small fraction of coronary artery anomalies with a reported prevalence of 0.017–0.03% based on angiography studies [8,25,38,39,43,44].

The LCA, in this case, ran between the pulmonary artery and the origin of the aorta and was “interarterial.” The acute angle run of the LCA (slit-like opening) at the origin and its “interarterial” position may have compromised the lumen and blood flow [41,45]. In addition, increased expansion of the aorta during exercise might occlude the already slit-like LCA opening by further compressing the orifice [41,45]. This results in acute myocardial ischemia, fatal arrhythmia, and sudden death [41]. Moreover, an “interarterial” position correlates with a higher incidence of angina, syncope, and sudden death [12,41]. In fact, “AAOLCA-IA” accounts for about one-third of the causes of sudden cardiac-related death in adolescent athletes and American recruits [46,47,48]. Furthermore, although rare, sudden deaths are not limited to adolescents during exercise, but have also been reported to occur during non-exercise in younger patients [30,48]. Therefore, when treating young patients with angina pectoris, myocardial infarction, or cardiac syncope, coronary angiography or cardiac multidetector computed tomography (MSCT) should be performed to determine the possibility of this abnormality [41]. MSCT is a useful test that is minimally invasive and can not only diagnose AAOLCA-IA, but also visualize plaque [48]. Furthermore, surgery is recommended for this course [49]. Other anatomic variant courses of abnormal LCA are considered benign [50].

For reference to coronary artery surgery, the inner diameter of the coronary artery origin and the length from the STJ were measured, and the branching of the coronary artery was examined. The inner diameter of the right Valsalva sinus of each coronary artery in this case was 4.5 mm of RCA, 2.5 mm of LCA, and 1 mm of CA, which was sufficiently large to accommodate a typical 1–3 mm diameter coronary catheter. In the case of aortic root surgery, the aortic incision is sometimes made relatively close to the STJ to ensure a good operative field [51]. In general, the average distance from the right and left coronary orifices to the STJ was 4.1 ± 3.4 mm or 5.4 ± 3.7 mm [51]. In the present case, the RCA, LCA, and CA were located below (caudal) the STJ, with distances from the STJ of 8.4, 6.8, and 5.1 mm, respectively. Although this case did not differ from the average distance, the distance from the STJ should be carefully considered intraoperatively to prevent coronary artery damage.

To the best of our knowledge, only three case reports of RCA, LCA, and CA originating from the right Valsalva sinus in AAOLCA were published, including this study [27,28]. Lipoff reported a case in which the RCA, LCA, and CA originated from the right Valsalva sinus, and the LCA was septal [28]. However, because of angiography in both cases, no details of RCA, LAD, or CA branching were shown [27,28]. In this case, the RCA, LCA, and CA originated from the right Valsalva sinus, and the LCA was an interarterial in origin. Because this was a dissected cadaveric case, the coronary arteries were observed in detail, except for the normal CA, LCA, and RCA branches. However, the three branches of LAD, LCx, and DB from the LCA were distinctive. Such details of coronary artery breaks are important in cardiac surgery. This is the first report of “AAOLCA-IA with CA” in a corpse.

One CA is reported to originate from the right sinus of Valsalva in 16.9–68.0% of cases [31,32,33,34,35]. In addition, 0.3% of cases have two CAs originating from the right sinus of Valsalva [45]. Anatomically, CBs are divided into two types: those that branch directly from the aorta and those that branch from the proximal right coronary artery, with the former being called the third coronary artery [52,53]. The frequency is said to range from 30.0% to 53.8% and is reported to occur more frequently in those with underlying diseases such as coronary artery disease and in males. Furthermore, RCA and CA originate separately from the right sinus of Valsalva in 12.6% of cases [54]. Because it is not a rare case, CA has not received much attention in anatomy textbooks [55]. However, evidence of the CB importance can be seen in neonatal and pediatric cases. Edwards [52] stated that the third coronary artery originating from the sinuses of Valsalva, including the CB, is more frequently found in adult hearts than in fetal hearts, which supports the concept that some coronary arterial patterns are not fully established at birth. Miyazaki [53] reported that the existence of multiple third coronary artery orifices and the fact that pathologic hearts have a higher incidence than normal hearts indicate that the third coronary artery develops and contributes to collateral circulation after birth. The CA often anastomoses with the branch of the LAD and forms the Vieussens’ arterial ring. This ring represents a significant path of the collateral bloodstream under coronary insufficiency conditions [56,57]. Schlesinger [58], in their study of 631 cadaveric hearts, reported that the extent of myocardial damage resulting from obstructed coronary arteries may be determined by the presence or absence of anastomosis between the CA and other arteries. Although the Vieussens’ ring with CB was not formed in this case, an opening of the CA may develop in the right Valsalva sinus after birth. In any case, the running of the CA and CB should continue to be noted.

## 5. Conclusions

This study revealed that the right Valsalva sinus had three openings observed in the cadaver of a 99-year-old Japanese man. The course and type of the coronary arteries were “interarterial” and “AAOLCA-IA with CA” and were observed for the first time in a cadaver. This report can contribute to preoperative evaluation and anatomy education during coronary artery surgery.

## Figures and Tables

**Table 1:** Reports of coronary arteries originating from the right sinus of Valsalva with three separate ostia.

Observation	Coronary Arteries	Proximal couse of LAD or LCA	Number of Cases	References
Angiography	RCA, LAD, LCx	Interarterial	One case	[7]
Angiography	RCA, LAD, LCx	Interarterial	One case	[14]
Angiography	RCA, LAD, LCx	Interarterial	One case	[15]
Angiography	RCA, LAD, LCx	Interarterial	One case	[16]
Angiography	RCA, LAD, LCx	Interarterial	One case	[17]
Angiography	RCA, LAD, LCx	Prepulmonary	One case	[18]
Angiography	RCA, LAD, LCx	Prepulmonary	One case	[19]
Angiography	RCA, LAD, LCx	Prepulmonary	Four cases	[20]
		Septal	Three cases	
Angiography	RCA, LAD, LCx	Septal	One case	[21]
Angiography	RCA, LAD, LCx	-	One case	[22]
Angiography	RCA, LAD, LCx	-	One case	[23]
Angiography	RCA, LAD, LCx	-	1 of 51 (2.0%)	[24]
Angiography	RCA, mLAD, aLAD	Prepulmonary (aLAD)	One case	[25]
Angiography	aRCA, pRCA, CA	-	One case	[26]
Angiography	RCA, LCA, CA	Retroaortic	One case	[27]
Angiography	RCA, LCA, CA	Septal	One case	[28]
Cadaver	RCA, LCA, CA	Interarterial	One case	Present study

aRCA, anterior right coronary artery; aLAD, accessory left anterior descending artery; pRCA, the posterior right coronary artery; CA, conus artery; LCx, left circumflex branch; LAD, left anterior descending artery; LCA, left coronary artery; mLAD, main left anterior descending artery; RCA, right coronary artery; -, unknown.

**Table 2:** Reports of conus arteries originating from the right sinus of Valsalva.

Observation	Coronary Arteries	Proximal Couse of LAD or LCA	Number of Cases	References
Angiography	RCA, RCA or CA	-	Two cases	[29]
Angiography	X, CA	-	14 of 83 (16.9%)	[30]
Angiography	X, CA	-	34 of 50 (68.0%)	[31]
Cadaver	X, CA	-	242 of 500 (48.4%)	[32]
Cadaver	X, CA	-	8 of 25 (32.0%)	[33]
Angiography	X, CA	-	154 of 700 (22.0%)	[34]
	X, CA, CA	-	2 of 700 (0.3%)	
Cadaver	RCA, CA RCA, CA, CA	-	12 of 95 (12.6%)	[35]
		-	1 of 95 (1.1%)	
Angiography	aRCA, pRCA, CA	-	One case	[26]
Angiography	RCA, LCA, CA	Retroaortic	One case	[27]
Angiography	RCA, LCA, CA	Septal	One case	[28]
Angiography	RCA, LAD, LCx, CA	Septal	One case	[36]
Angiography	RCA, LCD, LCx, CA	Septal	One case	[37]
	RCA (LCD), SAN, LCx, CA	Septal	One case	
Cadaver	RCA, LCA, CA	Interarterial	One case	Present study

aRCA, anterior right coronary artery; aLAD, accessory left anterior descending artery; pRCA, the posterior right coronary artery; CA, conus artery; LCx, left circumflex branch; LAD, left anterior descending artery; LCA, left coronary artery; mLAD, main left anterior descending artery; RCA, right coronary artery; X, third or two coronary artery; -, unknown.

## Data Availability

Data supporting the findings of this study are available from the corresponding author upon reasonable request.
